# Neural network model for prediction of possible sarcopenic obesity using Korean national fitness award data (2010–2023)

**DOI:** 10.1038/s41598-024-64742-w

**Published:** 2024-06-24

**Authors:** Jun-Hyun Bae, Ji-won Seo, Xinxing Li, SoYoung Ahn, Yunho Sung, Dae Young Kim

**Affiliations:** 1 Institute of Sports Science, Department of Physical Education, Seoul National University, Seoul, Republic of Korea; 2 Able-Art Sport, Department of Theory, Hyupsung University, Hwaseong, Gyeonggi-Do Republic of Korea; 3 Health and Exercise Science Laboratory, Department of Physical Education, Seoul National University, Seoul, Republic of Korea; 4 Senior Exercise Rehabilitation Laboratory, Department of Gerokinesiology, Kyungil University, Gyeongsan-Si, Gyeongsanbuk-Do Republic of Korea

**Keywords:** Sarcopenic obesity, Physical fitness, Hyperparameter, Sequential neural network, Older adult, Geriatrics, Weight management

## Abstract

Sarcopenic obesity (SO) is characterized by concomitant sarcopenia and obesity and presents a high risk of disability, morbidity, and mortality among older adults. However, predictions based on sequential neural network SO studies and the relationship between physical fitness factors and SO are lacking. This study aimed to develop a predictive model for SO in older adults by focusing on physical fitness factors. A comprehensive dataset of older Korean adults participating in national fitness programs was analyzed using sequential neural networks. Appendicular skeletal muscle/body weight was defined as SO using an anthropometric equation. Independent variables included body fat (BF, %), waist circumference, systolic and diastolic blood pressure, and various physical fitness factors. The dependent variable was a binary outcome (possible SO vs normal). We analyzed hyperparameter tuning and stratified K-fold validation to optimize a predictive model. The prevalence of SO was significantly higher in women (13.81%) than in men, highlighting sex-specific differences. The optimized neural network model and Shapley Additive Explanations analysis demonstrated a high validation accuracy of 93.1%, with BF% and absolute grip strength emerging as the most influential predictors of SO. This study presents a highly accurate predictive model for SO in older adults, emphasizing the critical roles of BF% and absolute grip strength. We identified BF, absolute grip strength, and sit-and-reach as key SO predictors. Our findings underscore the sex-specific nature of SO and the importance of physical fitness factors in its prediction.

## Introduction

Sarcopenic obesity (SO) is defined by combining the criteria for sarcopenia and obesity in older adults^1^. It has an increased prevalence in older adults and is associated with a high risk of disability, morbidity, and mortality^2–4^. Several defined SO criteria exist; SO based on appendicular skeletal muscle (ASM) and body weight (BW, kg) ratio is highly related to metabolic syndrome^5^ and accelerated physical performance decline^6^.

The SO prevalence is 6.1% among men and 7.3% among women and is associated with insulin resistance, inappropriate nutrition, and low physical activity^7^. Its sex-specific prevalence is 15.46% among men and 13.59% among women aged 75–84 years—the risk factors vary with sex^8^. Older women have a higher SO prevalence than older men (31.3% vs 19.6%)^9^. A high level of physical activity and proper diet^10^ are beneficial in decreasing SO prevalence among women^11^. Among 7161 subjects aged > 20 years, a lower self-reported physical functioning questionnaire score was associated with reduced skeletal muscle mass and SO^12^. SO is present in 7% of middle-aged women and is associated with poor physical performance^13^. It has a synergistic effect on physical function deterioration in older adults, with a more notable effect in women^14^. SO prevalence according to sex depends on physical performance results: it is necessary to determine how physical fitness factors influence SO.

Reportedly, high physical fitness levels are related to a decreased SO risk among older adults^15^. One of the sequential neural network results predicted sarcopenia, which is highly related to waist circumference (WC, cm), absolute grip strength (kg), and body fat (BF, %), to be related to SO risk^16^. SO, defined based on WC, is related to absolute grip strength, body composition, and low Short Physical Performance Battery scores^17,18^. WC, grip strength, and gait of older women also presented as SO diagnostic tools^19^. A systematic review and meta-analysis indicated that early diagnosis and appropriate interventions to reduce SO prevalence and various adverse outcomes are necessary^20^. SO is associated with reduced muscular strength and physical performance in older adults; however, studies on the relationship between SO and physical fitness factors are limited.

Recently, artificial intelligence techniques, including deep learning and machine learning, have gained attention for predicting many diseases, especially sarcopenia^21^. Many studies have focused on deep learning to define sarcopenia from chest X-ray^22–24^, and computed tomography images^25^, and machine learning to develop a sarcopenia prediction model from 10 different analysis models^26^. Overall, predictions based on sequential neural network SO studies and the relationship between physical fitness factors and SO are lacking. Therefore, our study aims to develop and validate a predictive model for SO in older adults using BF and absolute grip strength, assessed through a neural network analysis, to better understand how physical fitness factors contribute to the prediction of SO. We hypothesized that BF and absolute grip strength would have a significant impact on SO prediction.

## Materials and method

### Dataset and data collection

The dataset was approved by the Institutional Review Board (7,002,320–202,303-HR-001) and comprised physical fitness measurements of Korean adults aged ≥ 65 years, all of whom voluntarily participated at 75 national fitness centers as part of the Korean Physical Fitness Award project. Data were collected from January 2010 to March 2023 (*n* = 1,805,151; Supplementary Fig. S1). In the first data collection phase, 336,149 participants were excluded because of obesity without sarcopenia, sarcopenia without obesity, and null values. The second phase of data collection included columns with > 20% null and missing values removed and records of individuals aged < 64 years (*n* = 1,331,691). In the final data collection phase, 29,766 records were excluded based on interquartile range (IQR) outliers, which focused on univariate outlier detection due to the nature of our data and the specific neural network analysis applied. Outliers were defined as those exceeding the upper bound (Q3 + 1.5 × IQR) or falling below the lower bound (Q1 − 1.5 × IQR), to ensure the quality and reliability of the data analysis. The final dataset contained 107,545 participants. The data analysis environment was implemented on Windows 11 (× 64 version), with a 13th Gen Intel(R) Core (TM) i9-13900HX processor (2.20 GHz), 32 GB RAM, an NVIDIA GeForce RTX 4060 graphics processing unit, and Python (version 3.11.8) with TensorFlow (version 2.15.0).

### Define sarcopenic obesity

ASM was quantified and estimated using high-quality anthropometric formulas. ASM was calculated as 0.193 $\times$ Weight (kg) + 0.107 $\times$ Height (m)−4.157 $\times$ Sex (male = 1, female = 2)–0.037 $\times$ Age (years)– 2.631^27^. ASM/BW (%) was calculated by dividing the ASM value by BW (kg) and multiplying by 100^28^.

Possible SO—modified skeletal muscle mass index (SMI)^28^—was defined as ASM/BW < Me 2 standard deviations (SD) below the mean value of sex-specific young normal adults (reference group: 20–39 years)^7^. The cutoff points were 34.59% for men and 27.42% for women (Supplementary Fig. S2). WC was used as the Korean abdominal obesity criterion ($\ge$ 90 cm, men; $\ge$ 85 cm, women)^7,29,30^. We divided participants into two groups (possible SO and normal) and excluded those without sarcopenia and obesity.

### Independent and dependent variables

Independent variables included age, sex, height, weight, body mass index (BMI), body fat (BF), WC, systolic blood pressure (SBP), diastolic blood pressure (DBP), and physical fitness measurements such as sit-and-stand-up test (count), 2-min step test (count), time-up-and go test (TUG, s), figure of 8 walk test (s), absolute grip strength, and sit-and-reach^31^. The dependent variable was binary (i.e., normal vs possible). Trained physical fitness instructors followed all physical measures^32^. The sit-and-stand-up test quantified the participant's ability to transition from a seated to a standing position by recording the total number of complete cycles achieved within a 30-s interval. The 2-min step test quantitatively assessed the participants' physical endurance by recording the total number of in-place steps completed during a 2-min duration while standing. The time-up-and go test was measured as the time (second) to reach a target 3-m away and return. The figure-of-8 walk test involved setting cones at a triangle's base (3.6 m) and height (1.6 m), with a chair at the vertex. Participants started seated, navigated around both cones in succession, and returned to the chair for each lap, with the completion time (seconds) of two laps recorded. The absolute grip strength was measured using a handheld dynamometer (Takei, Niigata, Japan), with the participants gripping the handle and pulling with maximum force for 5 s at a 15° angle. The largest of the measurements obtained from both hands was recorded. The sit-and-reach test was measured to assess flexibility, with the distance (cm) measured on three times; the maximum value achieved was documented as the final score.

### Statistical modeling

#### Variance inflation factor, tolerance, and correlation between variables

The dataset included both independent and dependent variables. A constant term is added to the dataset to include an intercept in a regression model. The variance inflation factor (VIF) corresponding to each variable was calculated using the formula (1/1 − R^2^), where R^2^ is the variable regression coefficient with respect to all other variables. VIF assesses the extent to which the variance of an estimated regression coefficient is inflated owing to multicollinearity. A VIF > 10 is used to identify high multicollinearity^33^. Variables with VIF > 10 indicated significant multicollinearity. Tolerance was calculated as the VIF reciprocal (1/VIF). Tolerance < 0.1 indicated high multicollinearity^34^. VIF and tolerance measure the extent to which a variable is unexplained by other predictors in the model. Pearson’s correlation (r) was indicated by the pairwise correlation coefficient between ASM/BW and independent variables. The correlation threshold had an absolute value > 0.80^35^.

#### Data normalization and sampling

Data were normalized using *MinMaxScaler* to avoid over-reliance on certain features during speed learning, by restricting all variables to 0–1. Datasets were balanced via undersampling using *RandomUnderSampler* (random state = 42) by reducing oversampling between “normal” and “obese.” Data were split into training and test sets using a train–test split function (80 [training]:20 [test]).

#### Hyperparameter tunning

The study employed keras (v2.15.0) and keras tuner (v1.4.6) to refine a dataset using *Random Under Sampling* for class equilibrium. The neural network featured two dense layers with 32–300 units and dropout rates of 0–0.5, leading to a sigmoid output for binary classification. The learning rate choices of the *Adam* optimizer were 0.01, 0.001, and 0.0001. Hyperband tuning determined the best parameters, supplemented by *EarlyStopping* after five epochs without loss improvement. The optimized model for the balanced dataset showed the following essential hyperparameters: layer units, learning rate, dropout rate, and batch size^36–38^.

#### Sequential neural networks analysis

The dataset was split into training and validation sets using stratified K-fold (k = 5). The model setup ensured that each fold had the same proportion of *possible SO* classification labels (normal vs possible SO) as the original dataset. Training and validation sets were defined for each fold^39,40^.

This study used a sequential neural network model in which the first and second layers contained and were based on *ReLU* activation and the dropout layer with a rate from results of hyperparameter tuning. The input data included VIF, tolerance, and correlation thresholds. The final layer was a dense layer with a single unit and a “sigmoid” activation function suitable for binary classification (normal vs possible SO). The model was compiled with a binary cross-entropy loss function, the *Adam* optimizer with a learning rate that was used from results of hyperparameter tunning, and accuracy was used to monitor the training performance of the model^41,42^.

Two callbacks are used to monitor and control a training dataset. *EarlyStopping* monitored the validation loss, terminated training (patience = 20), and set weights of the model to those of the optimized model. *ModelCheckpoint* monitors validation loss and saves the model with the lowest validation loss during training^32,43^.

The model was trained using the training data for a maximum of 200 epochs, with a batch size based on results of hyperparameter tuning. The training process aims to minimize binary cross-entropy loss. If the validation loss did not improve for 20 consecutive epochs, the training was terminated and the model weights were set to the optimized state using the *EarlyStopping* callback. After training, the model with the optimized performance on the validation set was loaded with the *TensorFlow* and *Keras model* functions. These functions are stored in an optimized neural network model^32,44^.

#### Performance evaluation and prediction in the optimized model

The optimized model loaded from the previous step was used to predict the validation set. The predictions were obtained for each sample in the validation set. To convert probabilities generated by the model into binary predictions, a threshold of 0.5 was applied. If the predicted probability was > 0.5, the sample was classified as positive (obese); otherwise, it was classified as negative (normal). The model performance was evaluated using a validation set in terms of accuracy, precision, recall, F1 score, mean absolute error (MAE), and mean squared error (MSE). The calculated performance metrics (accuracy, precision, recall, and F1 scores) were used as validation sets for each fold. A confusion matrix (CF) was prepared using true negatives (TNs), false positives (FPs), false negatives (FNs), and true positives (TPs) for each fold^45–47^. After training, the precision-recall curve was plotted using probabilities predicted from the validation dataset for each fold. The area under the precision-recall curve (AUPRC)^48^, a summary metric measuring the performance of the model across all classification thresholds, was calculated. Finally, a code was used to visualize the precision-recall curve and AUPRC corresponding to each fold to determine the ability of the model to distinguish between normal and obese classes.

#### Model-agnostic algorithms (SHAP) used in the optimized model

This study evaluated the feature importance of each variable used in the model by applying SHapley Additive exPlanations (SHAP)^49^. SHAP analysis used *DeepExplainer*, which was designed for deep-learning models, and SHAP values were calculated using a scaled training dataset. SHAP values obtained after analysis represent the contribution of each feature to the prediction obtained from the model for each variable in the training set. The dependence of the SHAP plot uses the relationship between SHAP and actual values of features corresponding to each observation in the dataset.

### Ethical approval

Approval was obtained by the Research Ethics Committee of Hyupsung University (Approval no. 7002320–202,303-HR-001). The procedures used in this study adhere to the tenets of the Declaration of Helsinki. The studies were conducted in accordance with local legislation and institutional requirements.

### Informed consent

Written informed consent for participation was not required from the participants, their legal guardians, or next of kin in accordance with the national legislation and institutional requirements. The informed consent was waived by Hyupsung University (IRB approval no. 7002320–202,303-HR-001).

### Human and animal rights

Approval was obtained by the Research Ethics Committee of Hyupsung University (Approval No. 7002320–202,303-HR-001). The procedures used in this study adhere to the tenets of the Declaration of Helsinki. The studies were conducted in accordance with local legislation and institutional requirements.

## Results

### The results of VIF, tolerance and correlation

**Figure 1:**
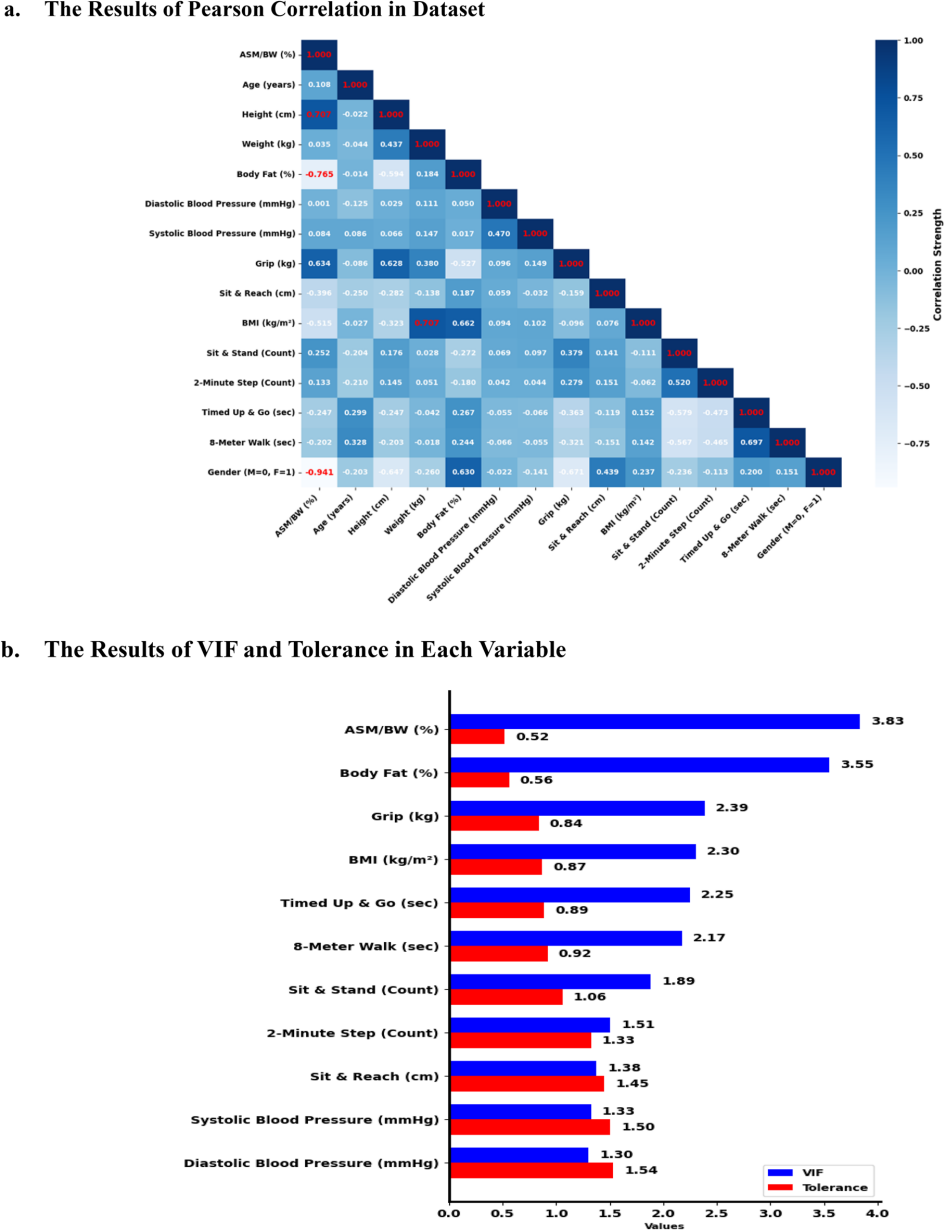
The Results of Correlation, VIF, Tolerance. (**a**) depicted a Pearson correlation (*r*) matrix heatmap showcasing the pairwise correlation coefficients between dependent variable (ASM/BW (%)) and independent variables (other variables). The scale to the right represents the correlation strength, with 1.0 indicating a perfect positive correlation, -1.0 indicating a perfect negative correlation, and 0 indicating no correlation. Deep blue squares denote strong positive correlations, while dark red squares indicate strong negative correlations. Lighter shades of blue and red represent weaker positive and negative correlations, respectively. The correlation threshold was an absolute value of over 0.70 (red color). (**b**) illustrated the VIF and Tolerance levels for different variables included in the regression analysis. The VIF values (Blue color), quantify the extent of multicollinearity in the regression model, with higher values indicating a greater degree of multicollinearity. Tolerance levels (Red color), were the inverse of VIF and indicate the proportion of variance of the predictor that was not explained by the other variables. This study had the threshold of he VIF above 5 or a Tolerance below 0.2, respectively. Abbreviation: VIF = Variance Inflation Factor.

The dependent variable ASM/BW was negatively correlated with BF (*r* =  −0.765) and sex (*r* =  −0.941) and positively with height (*r* = 0.707) (Fig. 1a). Age (*r* = 0.108), weight (*r* = 0.035), SBP (*r* = 0.001), DBP (*r* = 0.084), and BMI (*r* =  −0.515) correlated with ASM/BW. Physical fitness measurements, including absolute grip strength (*r* = 0.634), sit-and-reach (*r* =  −0.396), sit-and-stand-up (*r* = 0.252), 2-min step (*r* = 0.133), TUG (*r* =  −0.247), and figure of 8 walk (*r* =  −0.202) correlated with ASM/BW. Figure 1b illustrates low-to-moderate multicollinearity among ASM/BW (VIF = 0.52; tolerance = 1.92), BF (VIF = 0.56; tolerance = 1.79), absolute grip strength (VIF = 0.84; tolerance = 1.19), BMI (VIF = 0.87; tolerance = 1.15), TUG (VIF = 0.87; tolerance = 1.15), figure of 8 walk (VIF = 0.92; tolerance = 1.09), sit-and-stand-up (VIF = 1.06; tolerance = 0.94), 2-min step (VIF = 1.51; tolerance = 0.66), sit-and-reach (VIF = 1.38; tolerance = 0.72), SBP (VIF = 1.33; tolerance = 0.75), and DBP (VIF = 1.54; tolerance = 0.65). The ASM equation was based on age, height, weight, and sex; therefore, these variables were excluded. The input data for hyperparameter tuning and sequential neural network analysis included nine variables: BF, SBP, DBP, absolute grip strength, sit-and-reach, sit-and-stand-up, 2-min step, TUG, and figure of 8 walk.

### Final possible SO group in dataset

The proportion of women was higher than that of men (13.81% vs 0%) in the possible SO group (Supplementary Fig. S2a). The number of datasets was lower in the possible SO group (mean = 24.14, SD = 0.95) than in the other two groups including the excluded (mean = 27.02, SD = 3.50), normal (mean = 32.38, SD = 3.91) (Supplementary Fig. S2b). The final total number of datasets was 107,545 (possible SO, *n* = 27,964; normal, *n* = 79,581).

### Layers, drop rate, batch size, and learning rate in hyperparameter tunning

**Figure 2:**
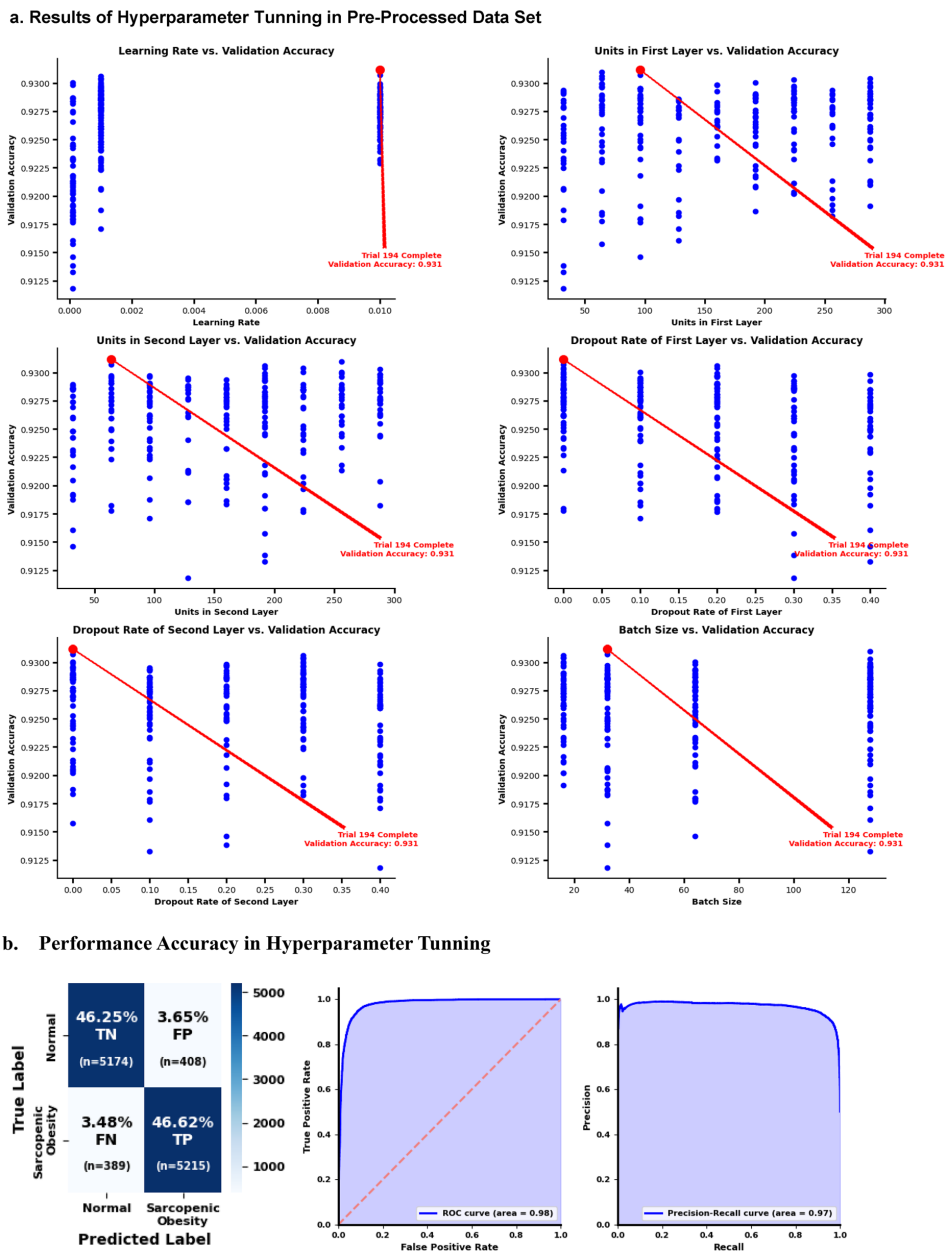

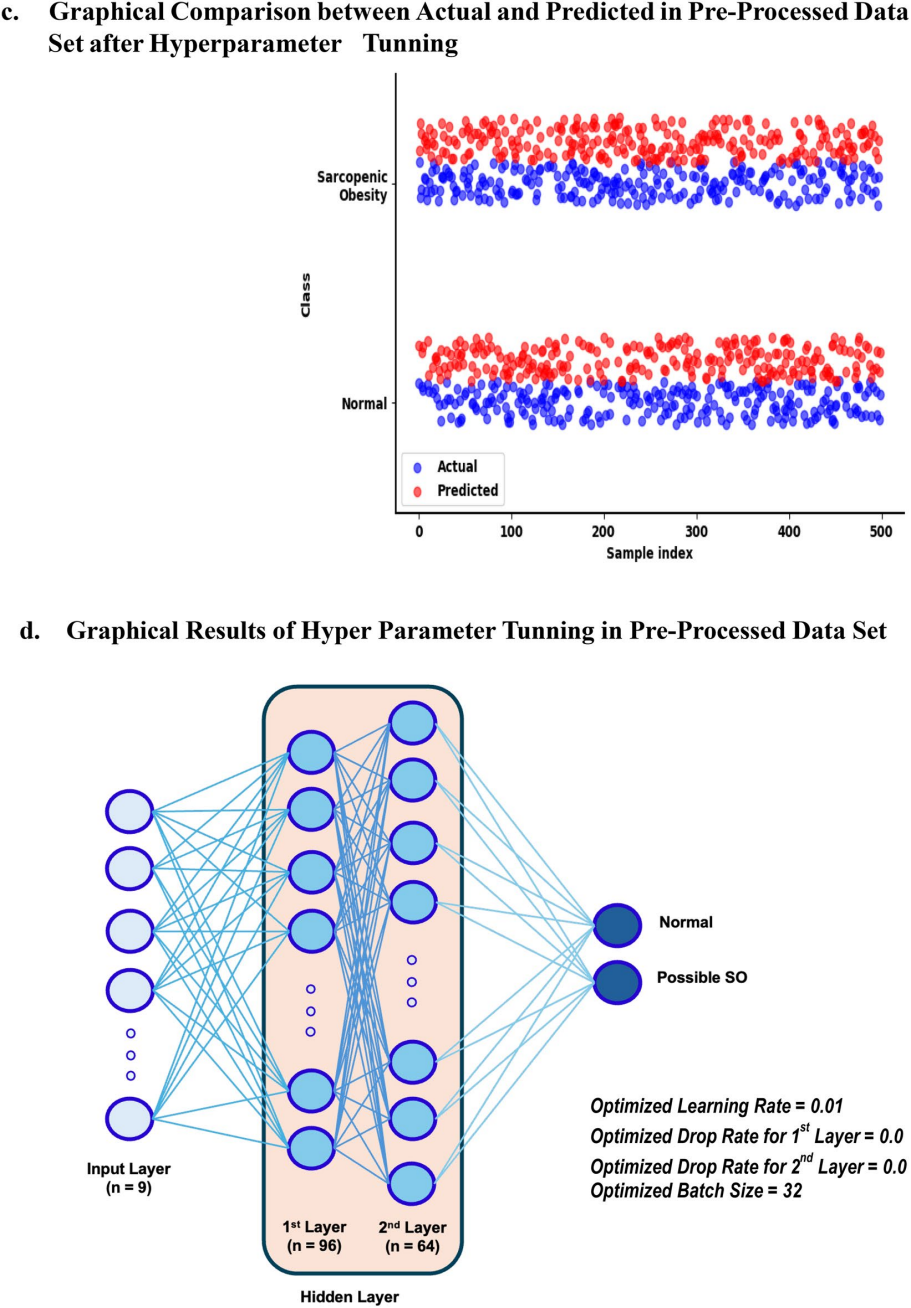
Results of Hyperparameter Tunning. This figure describes that emphasizing hyperparameter optimization for a neural network utilizing Keras, Keras Tuner, and imbalanced-learn. Initially, it delineates the dataset by isolating specific predictors of physical performance and a target variable, ‘Possible Sarcopenic Obesity', from a pre-processed Data Set (80–20 Split). (**a**) The results of hyperparameter tunning in pre-processed data set was based on learning rate, units of first layer, units in second layer, dropout rate of first layer, dropout rate of second layer, and batch size. (**b**) The graphical results of hyperparameter tunning in pre-processed data set was shown.

Hyperparameter tuning had the best trial completion (194), with a validation accuracy of 0.931 (Fig. 2). Figure 2a and d shows that the learning rate increased from 0.000 to 0.01. The best trial occurred at a learning rate close to 0.01, with a validation accuracy of approximately 0.931. The first and second layers of the neural network, 50–300, correlated with 96 and 64 units, respectively. The drop rates of the first and second layers were both 0.00 in the best trial with 0.931 validation accuracy. The batch size was 32 for the best 194 trials with 0.931 validation accuracy.

The performance of hyperparameter tuning was evaluated. The CF showed a TN of 46.25%; FP, 3.65%; FN, 3.48%; and TP, 46.62% (Fig. 2b). The precision-recall curve was 0.97 plotted using probabilities predicted using the validation dataset for hyperparameter tuning (Fig. 2b). The AUPRC was 0.98 (Fig. 2b). Graphical results of actual and predicted values in hyperparameter tuning show that the actual and predicted points overlapped, and that the best hyperparameter predictions aligned with actual labels. A good degree of overlap was noted in both classes (normal and possible SO), indicating a reasonable number of correct predictions by the model (Fig. 2c).

### The results of sequential deep learning analysis

**Figure 3:**
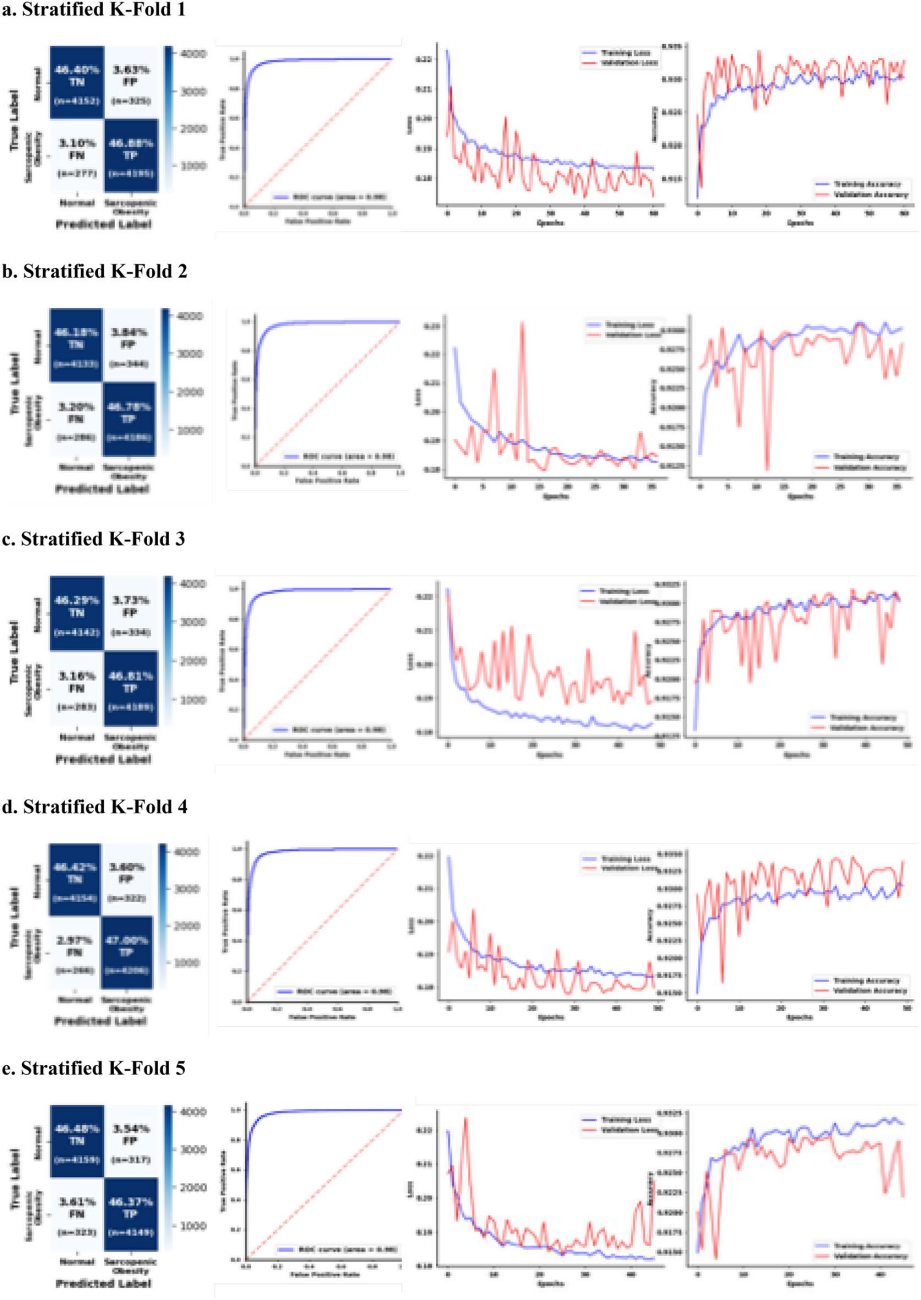

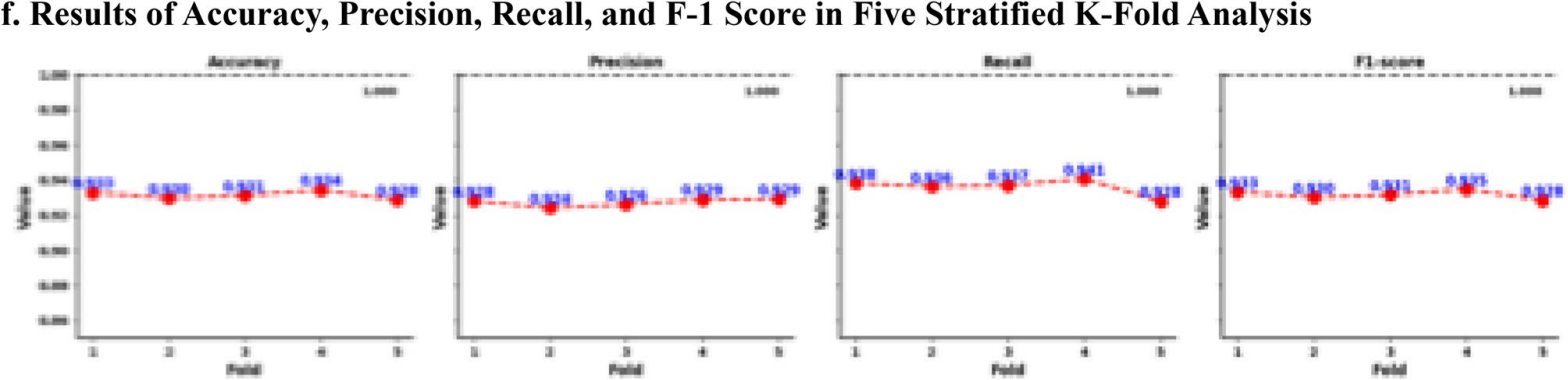
Results of Sequential Neural Network Analysis. (**a–e**) Stratified K-Fold cross-validation (k = 5) as a binary classification (0 = normal, 1 = possible sarcopenic obesity) dataset and the results are presented along with confusion matrices, ROC curves, and training and validation of accuracy and loss. (**f**) Performance metrics (ROC-AUC, accuracy, precision, recall, F1-score, MAE, and MSE) averaged across all folds to assess the overall model’s effectiveness. Abbreviations: TN = true negatives, FP = false positives, FN = false negatives, TP = true positives, Ave = average, ROC-AUC = receiver operating characteristic-area under curve, MAE = mean absolute error, and MSE = mean squared error.

The CF corresponding to Fold 1 (receiver operating characteristic [ROC] curve = 0.98, Precision-Recall Curve [PRC] = 0.977, Supplementary Fig. S3a) had the following values: TN (*n* = 4125, 46.09%), FN (*n* = 251, 2.80%), FP (*n* = 352, 3.52%), and TP (*n* = 4221, 47.17%) (Fig. 3a). The accuracy score was 0.933; precision, 0.923; recall, 0.944; and F1, 0.933. The neural network in the fold was stopped at 34 epochs using *callbacks* and *EarlyStopping* (Fig. 3a and f). The CF in Fold 2 (ROC curve = 0.98, PRC = 0.978, Supplementary Fig. S3b) had the following values: TN (*n* = 4090, 45.70%), FN (*n* = 247, 2.76%), FP (*n* = 387, 4.32%), and TP (*n* = 4225, 47.21%). The accuracy score was 0.929; precision, 0.916; recall, 0.945; and F1, 0.930. The neural network was stopped at 62 epochs using callbacks and EarlyStopping (Fig. 3b and f). The CF in Fold 3 (ROC curve = 0.98, PRC = 0.973, Supplementary Fig. S3c) had the following values: TN (*n* = 4153, 46.41%), FN (*n* = 283, 3.16%), FP (*n* = 323, 3.61%), and TP (*n* = 4189, 46.81%). The accuracy score was 0.935; precision, 0.928; recall, 0.937; and F1, 0.933. The neural network was stopped at 92 epochs using *callbacks* and *EarlyStopping* (Fig. 3c and f). The CF in Fold 4 (ROC curve = 0.98, PRC = 0.976, Supplementary Fig. S3d) had the following values: TN (*n* = 4140, 46.27%), FN (*n* = 246, 2.75%), FP (*n* = 336, 3.76%), and TP (*n* = 4226, 47.23%). The accuracy score was 0.935; precision, 0.926; recall, 0.945; and F1, 0.934. The neural network was stopped at 83 epochs using *callbacks* and *EarlyStopping* (Fig. 3d and f). The CF in Fold 5 (ROC curve = 0.98, PRC = 0.978, Supplementary Fig. S3e) had the following values: TN (*n* = 4132, 46.18%), FN (*n* = 284, 3.17%), FP (*n* = 344, 3.84%), and TP (*n* = 4188, 46.80%). The accuracy score was 0.930; precision, 0.924; recall, 0.936; and F1, 0.930. The neural network was stopped at 55 epochs using *callbacks* and *EarlyStopping* (Fig. 3e and f). In Fig. 3f, the overall stratified fivefold indicates that the average ROC curve was 0.979, and the MAE and MSE were 0.103 and 0.052, respectively. The average accuracy, precision, recall, and F1 scores were 0.931, 0.924, 0.941, and 0.932, respectively. Fold 1 was the optimized sequential neural network model in this study.

### The results of performance accuracy from optimized sequential neural network model

**Figure 4:**
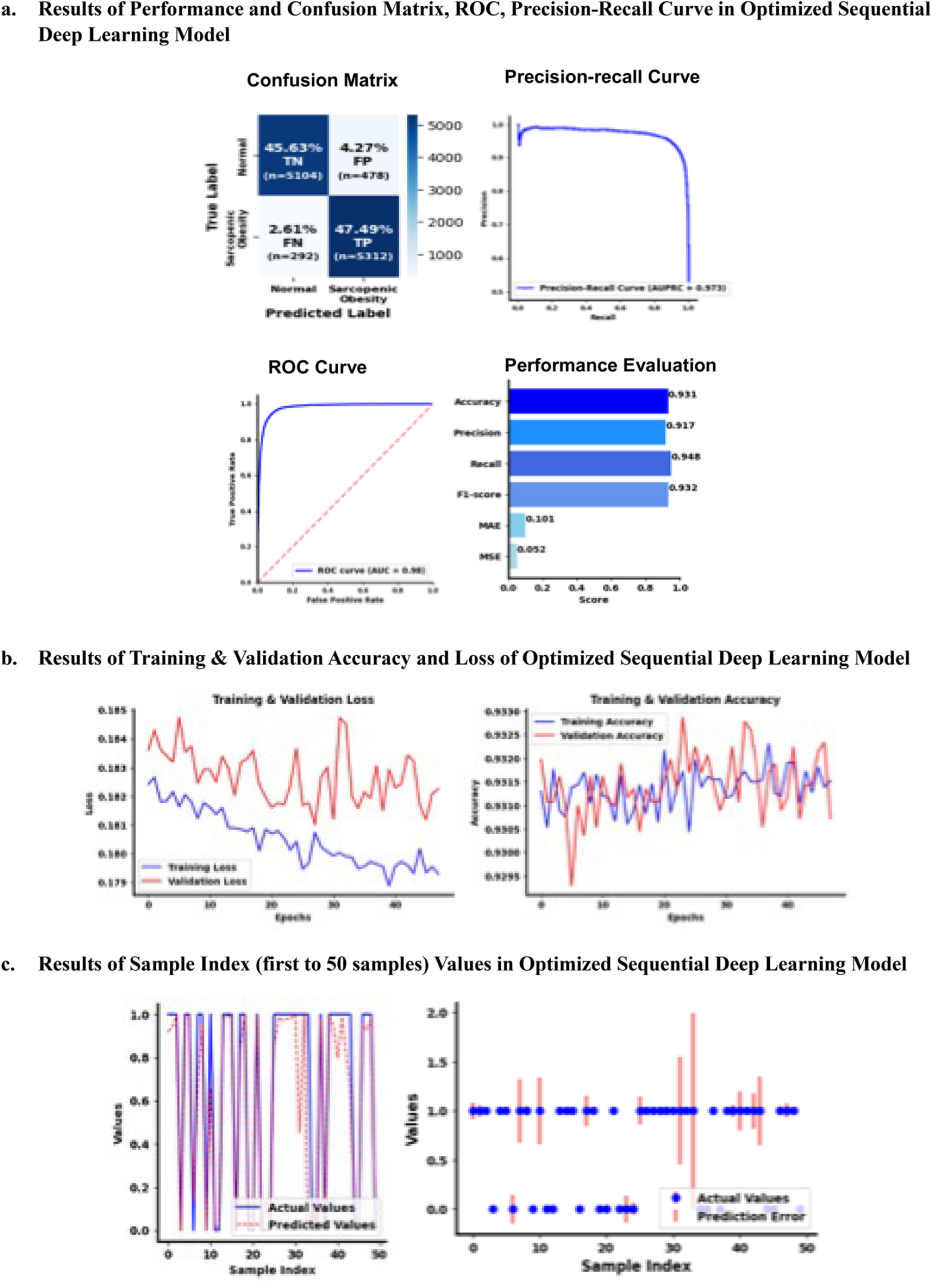
Results of Validation Accuracy from Optimized Sequential Deep Learning Model. (**a**) Evaluation of the best neural network model using a confusion matrix, accuracy, precision, recall, F1-score, MAE, MSE, precision-recall curve, and ROC curve. (**b**) Overfitting and underfitting are identified by comparing the loss and accuracy trends obtained using the training and validation datasets and the best neural network model. (**c**) Data are generated to compare the predicted and actual values (0 = Normal, 1 = possible SO), and a subset of these values is used to visualize the model’s prediction accuracy using the actual data for the first 50 samples. The prediction error is calculated by considering the absolute difference between the predicted and actual values for the first 50 data points, which represent the magnitude of the prediction error. The actual value with an associated error bar indicates the magnitude of the prediction error.

Fold 1 was the best optimized model. The CF of the best optimized sequential neural network model had the following values: TN (*n* = 5104, 45.63%), FN (*n* = 299, 2.67%), FP (*n* = 478, 4.27%), and TP (*n* = 5305, 47.43%). The performance accuracy (ROC curve = 0.98) in the best optimized sequential neural network model (maximum epochs = 200) showed accuracy, precision, recall, and F1 scores of 0.931, 0.917, 0.948, and 0.932, respectively (Fig. 4a). The MAE was 0.101 and MSE was 0.052. The best optimized model was stopped at 35 epochs using *callbacks* and *EarlyStopping* (Fig. 4b). The AUPRC was 0.971. Graphical results of the optimized sequential neural network for the 50-sample index had an MAE of 0.065 and MSE of 0.026 (R^2^ = 0.887; Fig. 4c). The prediction error is the difference between predicted values generated by the model and actual values, and lengths of the error bars indicate the magnitude of the error for each prediction. A longer bar indicates a larger error, and a shorter bar indicates a smaller error. Most predictions were close to actual values because the error bars were relatively short for most samples. However, a few samples had long error bars, indicating significant prediction errors for the 50-sample index.

### The results of SHAP analysis in optimized sequential neural network model

**Figure 5:**
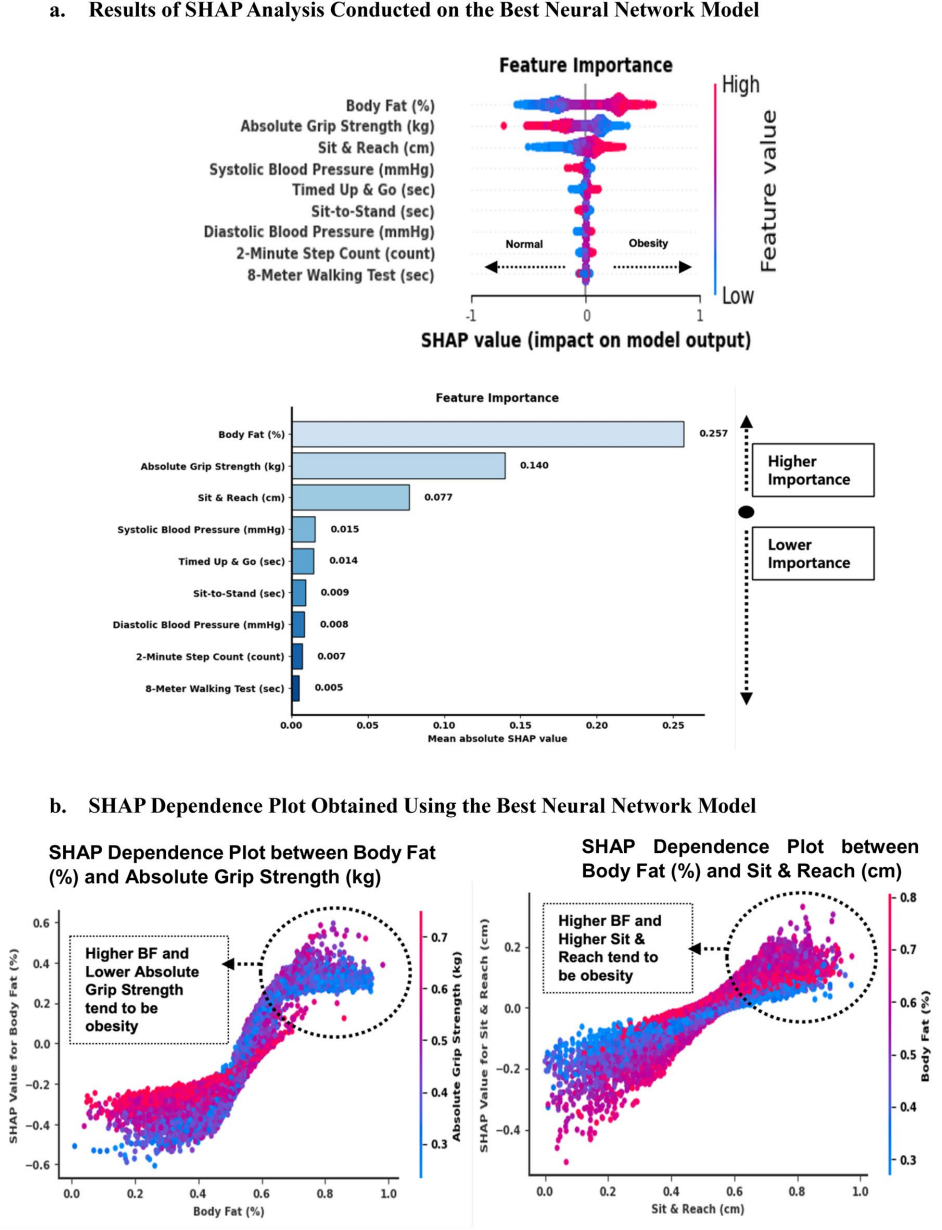

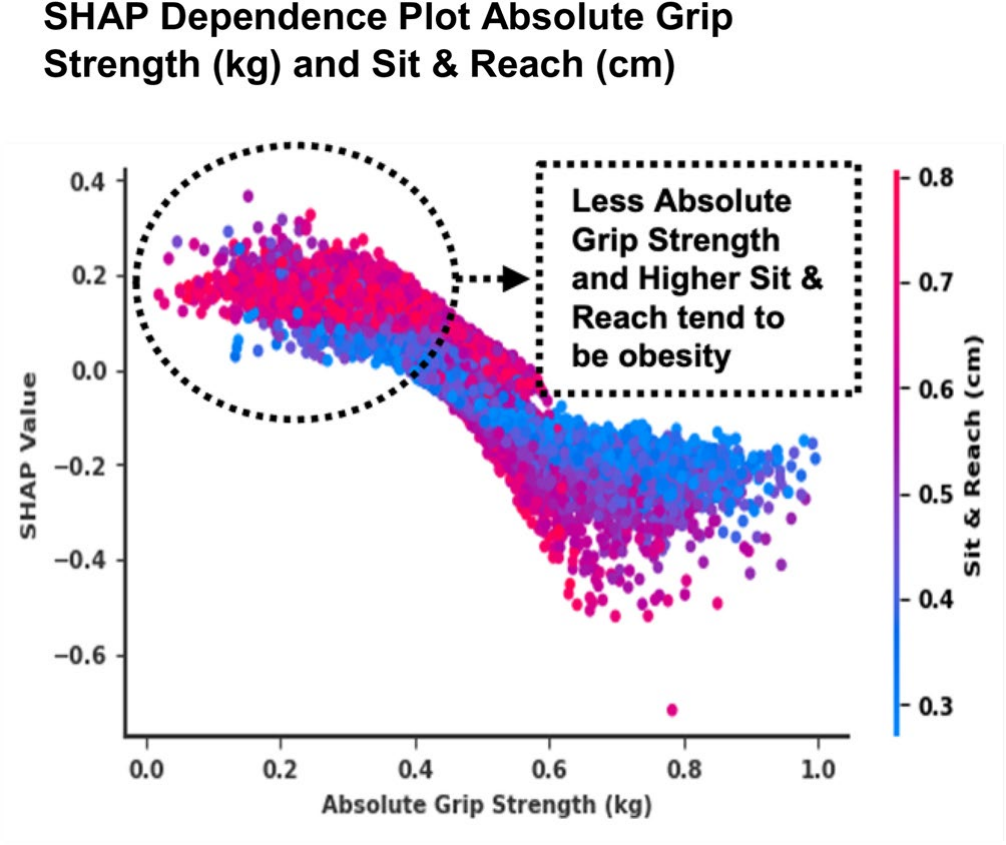
Results of Model-Agnostic Algorithms in Optimized Sequential Neural Network Model. (**a**) Detailed analysis of the SHAP feature importance obtained using the best neural network model, which computed SHAP values to represent the impact of each feature on the output of this model using the training dataset (blue: low and red: high). (**b**) Impact of body fat (%) and absolute grip strength (kg) on the predictions yielded by the optimized model. A SHAP dependence plot is used to analyze the relationship between body fat (%) and absolute grip strength (kg) and the SHAP values obtained from the optimized neural network model (blue: low and red: high). All values were based on the Minmax scaler dataset.

BF had the highest mean absolute SHAP value of 0.257, confirming its importance in the model’s decisions (Fig. 5). Absolute grip strength and sit-and-reach were the second and third most important features with mean absolute SHAP values of 0.140 and 0.077, respectively. Thus, BF and absolute grip strength were the most influential features in model predictions. Sit-and-reach contributed moderately to the model’s decisions.

The SHAP dependence plot showed that BF increased and SHAP values also increased non-linearly, suggesting that a higher BF had a stronger positive impact on the model’s output for predicting possible SO (Fig. 5b). Decreased absolute grip strength and increased BF were associated with possible SO. These results were related to lower values of absolute grip strength, and the impact of BF on the model’s output increased, indicating a potential interaction (Spearman’s correlation =  −0.610, *p* < 0.001) between these two features. The SHAP dependence plot between sit-and-reach and BF showed that SHAP values increased as sit-and-reach values increased, indicating a positive correlation with the model’s prediction. Individuals with a higher BF tended to have higher SHAP values for sit-and-reach, suggesting that BF interacted with flexibility as captured by sit-and-reach to influence the possible SO prediction (Spearman’s correlation = 0.259, *p* < 0.001). The SHAP dependence plot between absolute grip strength and sit-and-reach showed that decreased absolute grip strength and increased sit-and-reach values were associated with high SHAP values to predict possible SO (Spearman’s correlation =  −0.217, *p* < 0.001).

## Discussion

This study found a significantly higher prevalence of possible SO among women (13.81%), with no man exceeding the threshold. Hyperparameter tuning of the neural network resulted in a high validation accuracy of 93.1% with the best model configuration, including 96 and 64 units in the first and second layers, respectively; a learning rate of nearly 0.01; and a batch size of 32 (Fig. 2). The best optimized sequential neural network model was obtained from the stratified K-fold results (TN = 45.63%, FN = 2.67%, FP = 4.27%, TP = 47.43%, and ROC curve = 0.98) (Fig. 3). The SHAP analysis confirmed the importance of BF, absolute grip strength, and sit-and-reach in predicting possible SO, with BF emerging as the most significant feature (mean absolute SHAP value = 0.257) and demonstrating strong interactions with absolute grip strength, particularly affecting the model’s output on possible SO prediction (Fig. 5).

This study indicates that the possible SO of ASM/BW was based on 2 SDs below the mean value of sex-specific young normal adults, as cutoff points were 34.59% for men and 27.42% for women (mean = 24.14, SD = 0.95, Supplementary Fig. S2b). Reportedly, SO prevalence varies depending on the definition used, with higher rates observed when sarcopenia is defined by the ratio of ASM to BW rather than height squared^30^. Compared with those in a previous study, the cutoff point (29.9% for men and 25.1% for women; one SD below the mean value of sex-specific young normal adults) was less than our values because our criteria were based on two SDs below the mean value of sex-specific young normal adults. Further, possible SO prevalence was 13.81% among women and 0% among men (Supplementary Fig. S2a), consistent with the finding that SO prevalence among women increased with age (over 65 years) after adjusting for BW^7–9^. Thus, the prevalence and definition of possible SO differed according to sex and cutoff points for women.

This study showed that BF was highly associated with possible SO prediction (Fig. 5a). These results were related to SO, as defined using the ASM/BW ratio, and were more closely associated with metabolic syndrome than with sarcopenia or obesity alone^30^. Possible SO prevalence was higher among women than among men, suggesting that sarcopenia and SO in women were associated with an increased all-cause mortality risk independent of obesity, although this association was not significant in men^50^. Lower BF levels were reported in women with sarcopenia in a previous study, and our finding of a high BF level was related to possible SO^32,51^. The probability of possible SO had the highest feature importance factor of BF because obese adipose tissue inflammation dominates skeletal muscle inflammation, leading to SO, which is exacerbated by the age-related decline in muscle mass and function^52^. Moreover, SO significantly affects the physical performance of older adults (e.g., absolute grip strength) and increases their BF^53^. The biomarkers were not measured in this study; therefore, results of possible SO groups were different.

Our results also showed the second highest impact on the possible SO prediction model (Fig. 5a), suggesting that SO was associated with accelerated decline in physical fitness and increased risk of cardiometabolic diseases, and mortality, leading to its identification in aging^6^. A previous study also indicated that grip strength positively correlated in older women to identify SO^54^. Our results showed a similar pattern of low absolute grip strength, which was highly predicted for women with possible SO (Fig. 5a). The possibility of low grip strength was associated with elevated levels of pro-inflammatory cytokines, which decreased muscle strength and developed SO^55^. Using the same dataset as the Korean National Physical Fitness Award between 2010 and 2023, the sequential deep learning model showed that absolute grip strength was the most important factor for predicting possible sarcopenia in older adults^32^. The results of the deep learning model had high accuracy (AUCRC = 0.95) and recall with possible sarcopenia, and our results also showed that the possible SO prediction model had a high accuracy of performance (AUCRC = 0.97). Thus, this result supports that possible female SO with absolute grip strength is also as valid and easy to detect as SO and sarcopenia^56^.

Moreover, when determining the best machine learning for analyzing older adults’ physical fitness data, grip strength and walking ability (figure of 8 walk test) were closely associated with predicting grip strength in older adults^16^. However, our study showed that sit-and-reach had a high impact on possible female SO prediction (Fig. 5b), and the low level of absolute grip strength and high level of sit-and-reach tended to predict possible female SO (SHAP value increased to one; Fig. 5b). These results indicate the possibility that Korean older adults showed better fitness performance in all fitness tests, while having a higher BMI and BF^57^ than those in Western^58,59^ and other Asian countries^60^. Moreover, older women with a lower BMI have better muscular strength and flexibility^61^. Among 877 older adults, a population-based cross-sectional study showed that those with sarcopenia classified based on weight-adjusted SMI had a higher BMI, BF, and lower grip strength; therefore, results differed in terms of obesity indicators^62^. To the best of our knowledge, our results suggest that possible female SO had a high impact on the sit-and-reach score because we used SMI-adjusted weight, and possible SO was highly prevalent among women.

## Limitations

First, a significant prevalence of SO was identified only among women. This limitation restricts generalizability of our findings across sexes and suggests the need for further research, including a male cohort, to fully understand SO’s impact. Second, the study did not use gold-standard measurements of muscle mass such as dual-energy X-ray absorptiometry (DXA) to define ASM but relied on anthropometric formulas. Although these formulas are useful for large-scale studies, DXA provides more accurate measurements of muscle mass and can alter SO identification. Third, our analysis did not account for variables, such as physical activity level and nutritional intake, which are known to significantly influence muscle mass and obesity. The omission of these factors may limit our understanding of their contributions to SO and their outcomes, potentially overlooking key predictive or mitigating factors. Fourth, the risk of overfitting limits the predictive model developed. Despite employing hyperparameter tuning and validation techniques to optimize model performance, the complexity of neural networks inherently carries the risk of overfitting. This risk could limit the applicability of the model to external datasets or real-world scenarios, suggesting the need for further validation and testing of independent datasets to ensure its robustness and generalizability. Moreover, the removal of outliers using the IQR method aims to eliminate extreme values that might distort the predictive modeling process. While this approach helps in stabilizing the statistical analyses, it could potentially limit the generalizability of our findings to populations that exhibit extreme values. We are aware of this limitation and suggest that future studies could explore the impact of including more diverse population data to validate the robustness and applicability of our model across different clinical settings.

## Conclusion

This study highlighted the significant SO prevalence among older women (13.81%), with no observed prevalence among men, emphasizing the sex-specific SO nature. Through rigorous hyperparameter tuning, a highly accurate neural network model was developed, with a validation accuracy of 93.1%. This model featured a configuration of 96 units in the first layer and 64 units in the second layer, with an optimal learning rate close to 0.01 and a batch size of 32. The stratified K-fold validation approach underscored the effectiveness of the model, with a notable ROC curve value of 0.98, illustrating its robust predictive capability. Importantly, SHAP analysis identified BF, absolute grip strength, and sit-and-reach as key SO predictors. BF and absolute grip strength played a pivotal role in predicting possible SO in older women, significantly outperforming other metrics such as sit-and-reach in a developed neural network model with high validation accuracy. SO prevalence was exclusively observed in women, with a notable absence in men, underscoring sex-specific differences in SO manifestations. This study’s insights into sex-related nuances of SO prevalence and the impact of BF, absolute grip strength, and sit-and-reach offer a valuable perspective for future research and clinical practice in addressing SO complexity. Future research should focus on gender-related nuances in SO prevalence and explore targeted interventions based on physical fitness to mitigate SO’s impact. Also, the cutoff value of absolute grip strength and BF consider defining those values for specific implementation of clinical practice. In addition, our study design was cross sectional, which limited our ability to infer causal relationships between physical fitness factors and SO. Future research could implement a longitudinal design to track changes over time, providing insights into the causal mechanisms and the dynamic nature of SO development in older populations.

### Supplementary Information

Supplementary Information.

## Data Availability

All data generated during the current study have been included in this published article and its original dataset and coding file are available from the corresponding and first author (Dae Young Kim & Jun-Hyun Bae) on reasonable request.
