# Supplementary material for: Neural network model for prediction of possible sarcopenic obesity using Korean national fitness award data (2010–2023)
Source: Sci Rep. 2024 Jun 24;14:14565. doi: 10.1038/s41598-024-64742-w (PMC11196656; doi:10.1038/s41598-024-64742-w)
Supplement: Supplementary file 1 — Supplementary Information. [file 41598_2024_64742_MOESM1_ESM.pptx]

## Slide 1
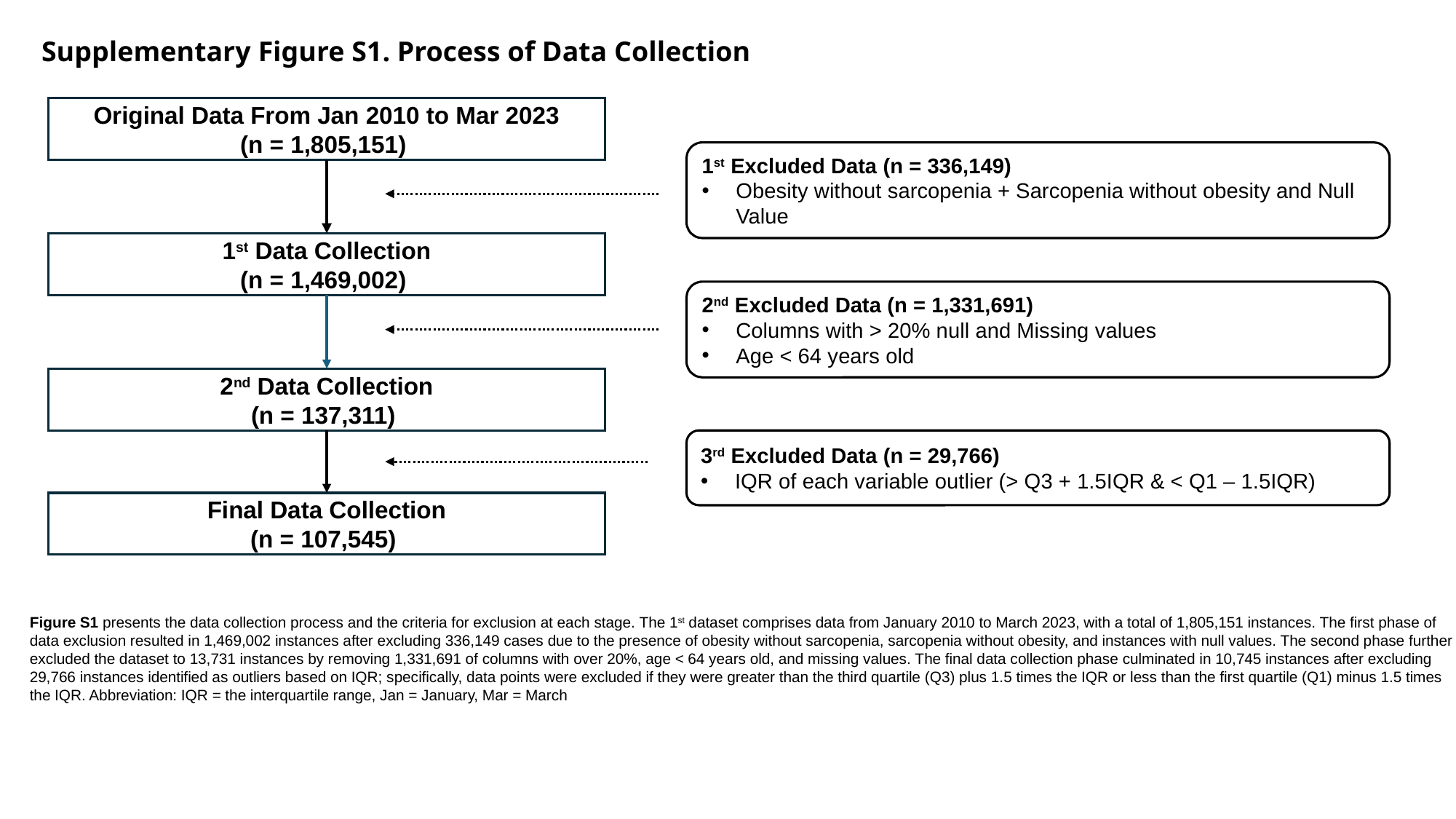

Supplementary Figure S1. Process of Data Collection
Original Data From Jan 2010 to Mar 2023
(n = 1,805,151)
1st Excluded Data (n = 336,149)
Obesity without sarcopenia + Sarcopenia without obesity and Null Value
1st Data Collection
(n = 1,469,002)
2nd Excluded Data (n = 1,331,691)
Columns with > 20% null and Missing values
Age < 64 years old
2nd Data Collection
(n = 137,311)
Final Data Collection
(n = 107,545)
Figure S1 presents the data collection process and the criteria for exclusion at each stage. The 1st dataset comprises data from January 2010 to March 2023, with a total of 1,805,151 instances. The first phase of data exclusion resulted in 1,469,002 instances after excluding 336,149 cases due to the presence of obesity without sarcopenia, sarcopenia without obesity, and instances with null values. The second phase further excluded the dataset to 13,731 instances by removing 1,331,691 of columns with over 20%, age < 64 years old, and missing values. The final data collection phase culminated in 10,745 instances after excluding 29,766 instances identified as outliers based on IQR; specifically, data points were excluded if they were greater than the third quartile (Q3) plus 1.5 times the IQR or less than the first quartile (Q1) minus 1.5 times the IQR. Abbreviation: IQR = the interquartile range, Jan = January, Mar = March

## Slide 2
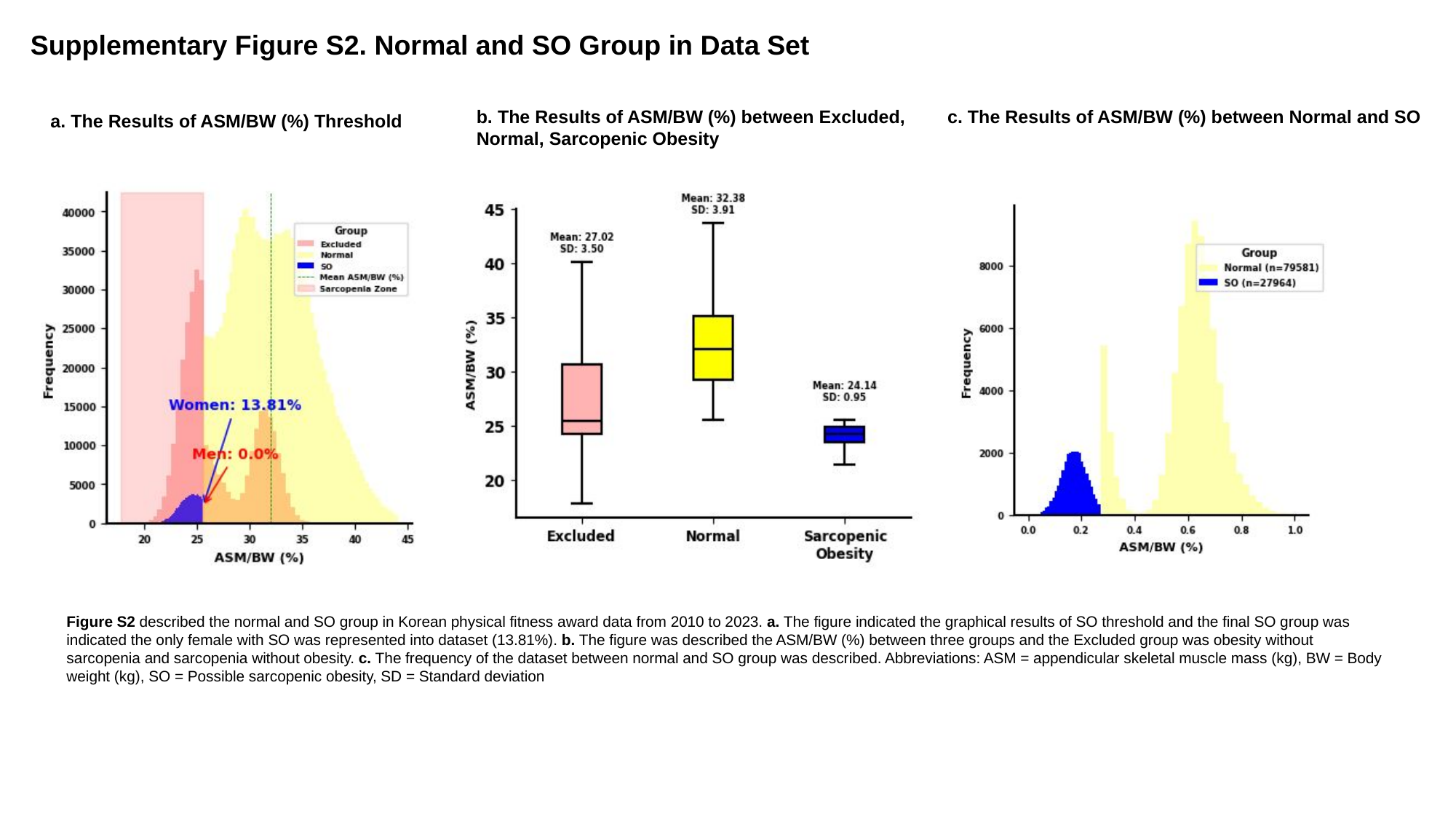

Supplementary Figure S2. Normal and SO Group in Data Set
b. The Results of ASM/BW (%) between Excluded, Normal, Sarcopenic Obesity
c. The Results of ASM/BW (%) between Normal and SO
a. The Results of ASM/BW (%) Threshold
Figure S2 described the normal and SO group in Korean physical fitness award data from 2010 to 2023. a. The figure indicated the graphical results of SO threshold and the final SO group was indicated the only female with SO was represented into dataset (13.81%). b. The figure was described the ASM/BW (%) between three groups and the Excluded group was obesity without sarcopenia and sarcopenia without obesity. c. The frequency of the dataset between normal and SO group was described. Abbreviations: ASM = appendicular skeletal muscle mass (kg), BW = Body weight (kg), SO = Possible sarcopenic obesity, SD = Standard deviation

## Slide 3
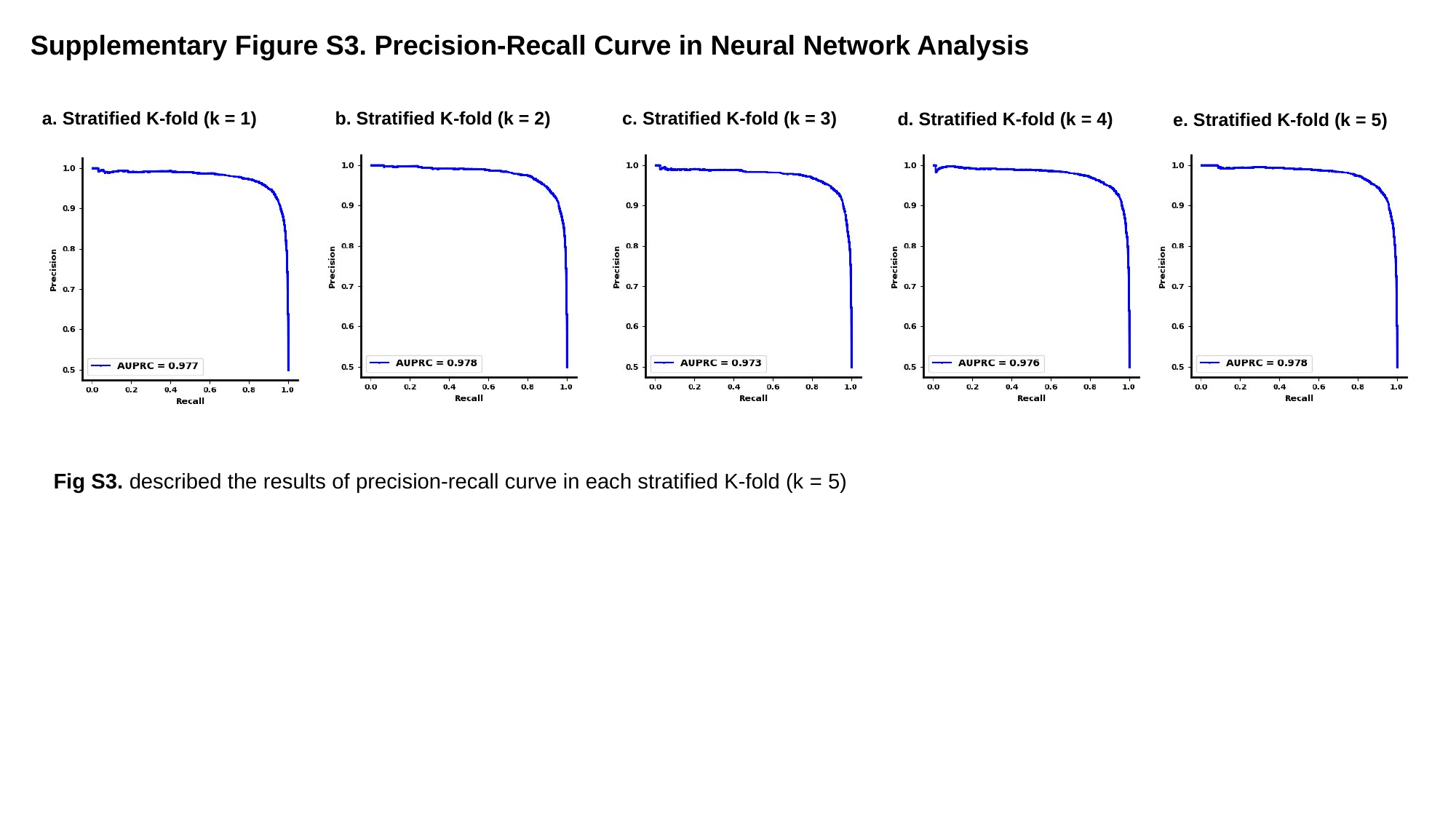

Supplementary Figure S3. Precision-Recall Curve in Neural Network Analysis
c. Stratified K-fold (k = 3)
b. Stratified K-fold (k = 2)
a. Stratified K-fold (k = 1)
d. Stratified K-fold (k = 4)
e. Stratified K-fold (k = 5)
Fig S3. described the results of precision-recall curve in each stratified K-fold (k = 5)
